# *Chaetomium* and *Chaetomium*-like Species from European Indoor Environments Include *Dichotomopilus finlandicus* sp. nov.

**DOI:** 10.3390/pathogens10091133

**Published:** 2021-09-03

**Authors:** Orsolya Kedves, Sándor Kocsubé, Teodóra Bata, Maria A. Andersson, Johanna M. Salo, Raimo Mikkola, Heidi Salonen, Attila Szűcs, Alfonz Kedves, Zoltán Kónya, Csaba Vágvölgyi, Donát Magyar, László Kredics

**Affiliations:** 1 Department of Microbiology, Faculty of Science and Informatics, University of Szeged, Közép fasor 52, H-6726 Szeged, Hungary; kedvesorsolya91@gmail.com (O.K.); kocsube@bio.u-szeged.hu (S.K.); bata.teodora1997@gmail.com (T.B.); a.szunyi@windowslive.com (A.S.); csaba@bio.u-szeged.hu (C.V.); 2 Department of Civil Engineering, Aalto University, P.O. Box 12100, FI-00076 Aalto, Finland; maria.a.andersson@helsinki.fi (M.A.A.); johanna.72salo@gmail.com (J.M.S.); raimo.mikkola@aalto.fi (R.M.); heidi.salonen@aalto.fi (H.S.); 3 Department of Applied and Environmental Chemistry, Faculty of Science and Informatics, University of Szeged, Rerrich Béla tér 1, H-6720 Szeged, Hungary; kedvesalfonz@yahoo.com (A.K.); konya@chem.u-szeged.hu (Z.K.); 4 National Public Health Center, Albert Flórián út 2-6, H-1097 Budapest, Hungary; magyar.donat@gmail.com

**Keywords:** *Chaetomium*, *Dichotomopilus*, indoor environment, extracellular enzymes

## Abstract

The genus *Chaetomium* is a frequently occurring fungal taxon world-wide. *Chaetomium* and *Chaetomium*-like species occur in indoor environments, where they can degrade cellulose-based building materials, thereby causing structural damage. Furthermore, several species of this genus may also cause adverse effects on human health. The aims of this research were to identify *Chaetomium* and *Chaetomium*-like strains isolated from indoor environments in Hungary and Finland, two geographically distant regions of Europe with drier and wetter continental climates, respectively, and to study their morphological and physiological properties, as well as their extracellular enzyme activities, thereby comparing the *Chaetomium* and *Chaetomium*-like species isolated from these two different regions of Europe and their properties. *Chaetomium* and *Chaetomium*-like strains were isolated from flats and offices in Hungary, as well as from schools, flats, and offices in Finland. Fragments of the translation elongation factor 1α (*tef1α*), the second largest subunit of RNA polymerase II (*rpb2*) and β-tubulin (*tub2*) genes, as well as the internal transcribed spacer (ITS) region of the ribosomal RNA gene cluster were sequenced, and phylogenetic analysis of the sequences performed. Morphological examinations were performed by stereomicroscopy and scanning electron microscopy. Thirty-one *Chaetomium* sp. strains (15 from Hungary and 16 from Finland) were examined during the study. The most abundant species was *Ch. globosum* in both countries. In Hungary, 13 strains were identified as *Ch. globosum*, 1 as *Ch. cochliodes,* and 1 as *Ch. interruptum*. In Finland, 10 strains were *Ch. globosum,* 2 strains were *Ch. cochliodes*, 2 were *Ch. rectangulare*, and 2 isolates (SZMC 26527, SZMC 26529) proved to be representatives of a yet undescribed phylogenetic species from the closely related genus *Dichotomopilus*, which we formally describe here as the new species *Dichotomopilus finlandicus*. Growth of the isolates was examined at different temperatures (4, 15, 20, 25, 30, 37, 35, 40, and 45 °C), while their extracellular enzyme production was determined spectrophotometrically.

## 1. Introduction

*Chaetomium* Kunze (Ascomycota, Sordariales) is the largest genus of the family Chaetomiaceae, present in various substrates and geographical regions [1]. More than 400 *Chaetomium* species have been described. The type species is *Ch. globosum* Kunze [2]. As cellulose-degrading fungi they possess the ability to degrade wet cellulosic building materials such as wood and plywood and synthetic building materials such as plastics and drywall [3]. Wet building materials are dominated by colonization and forming a dense mycelium covering most of the building materials, thereby damaging the building structure [4,5]. In addition to colonizing building materials, members of the genus *Chaetomium* are also known to produce more than 500 bioactive metabolites [6]. *Chaetomium globosum*, producing various mycotoxins—such as chaetoglobosin, chaetomin, chaetomugilin, and chaetoviridine—is the most common species of the genus in indoor environments [5,7].

Several *Chaetomium* species have been described to cause onychomycosis [8,9,10,11,12], skin surface infections [13], and cerebral mycosis [14,15,16]. Among them, some are able to opportunistically cause systemic infections and trigger severe allergic reactions that increase the risk of developing asthma [7]. On the other hand, some species of the genus *Chaetomium* are plant endophytes [17,18,19,20], producing high levels of various enzymes [21,22,23,24], antioxidants [25,26], as well as antifungal [25,27,28,29,30,31], antibacterial [28,32,33,34,35,36,37], and nematicidal metabolites [38,39], possessing anticancer [26,28,30,40,41,42,43,44,45,46,47,48,49,50,51] and anti-inflammatory activities [39,52], and being able to biosynthesize several types of nanoparticles [44,53,54]. The genus includes psychrotolerant [55], mesophilic, thermotolerant, and thermophilic species [5], which are widely used in the medicinal and industrial field (e.g., food, textile, and fermentation industries), agriculture (as biocontrol agents and in agricultural waste degradation), and waste processing (composting) [56]. 

The genus *Chaetomium* is generally characterized by rounded, ovoid, or obovate ostiolate ascomata covered with characteristic hairs. The walls of the ascomata are usually *textura intricata* (tissue of interwoven irregularly disposed hyphae with distinct interhyphal spaces, the walls not united), but they occur in *textura angularis* (tissue of short polyhedral cells without intercellular spaces). Ascomatal hairs can be straight (seta-like), flexible, curved, wavy, circulating, spirally curled, or otherwise branched in various morphologies. Asci are clavate or fusiform with 8 biseriate or irregularly arranged ascospores, evanescent. Ascospores are limoniform to globose, or irregular in a few species, bilaterally flattened, usually more than 7 μm in length. Asexual morphs, if present, are *Acremonium*-like [7].

*Chaetomium* is an intensively studied fungal genus worldwide, as it causes one of the biggest problems in indoor environments with damaging effects both to buildings and human health [3,4,7,54,57,58,59,60,61]. The closely related genus *Dichotomopilus* was first described by Wang et al. [7]; until then, members of the genus *Dichotomopilus* belonged to the genus *Chaetomium*. The genus name *Dichotomopilus* refers to the shape of terminal hairs of the ascomata, which are usually dichotomously branched. So far, this genus has included 12 species.

To the best of our knowledge, the diversity of this group of fungi has not yet been studied in Hungary. This study aimed to identify *Chaetomium* and *Chaetomium*-like strains isolated from Hungary and Finland, two geographically distant regions of Europe with drier and wetter continental climates, respectively, to study their morphology, determine their physiological properties, measure their extracellular enzyme activities, and compare the properties of *Chaetomium* and *Chaetomium*-like strains isolated from different sites of the two regions.

## 2. Results

**Figure 1:**
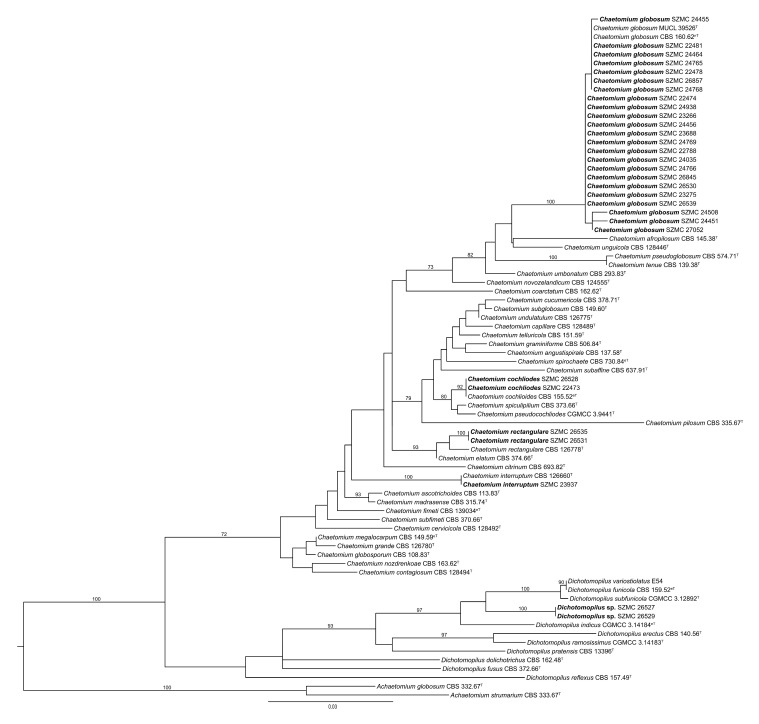
Maximum likelihood phylogeny of the examined isolates (set in bold) based on translation elongation factor 1α. T = ex-type, nT = ex-neotype, eT = ex-epitype. Numbers above branches are bootstrap values. Only values greater than 70% are shown.

Based on *tef1α* sequences, the most abundant species in this study was *Ch. globosum* in both countries (Table 1, Figure 1). In Hungary, 13 strains were identified as *Ch. globosum*, 1 as *Ch. cochliodes*, and 1 as *Ch. interruptum*, while in Finland, 10 strains were *Ch. globosum*, 2 strains were *Ch. cochliodes*, 2 were *Ch. rectangulare*, and 2 isolates (SZMC 26527, SZMC 26529) proved to be representatives of a yet undescribed phylogenetic species from the closely related genus *Dichotomopilus* (Figure 1).

### 2.1. Morphological Features of the Isolated *Chaetomium* Strains

**Figure 2:**
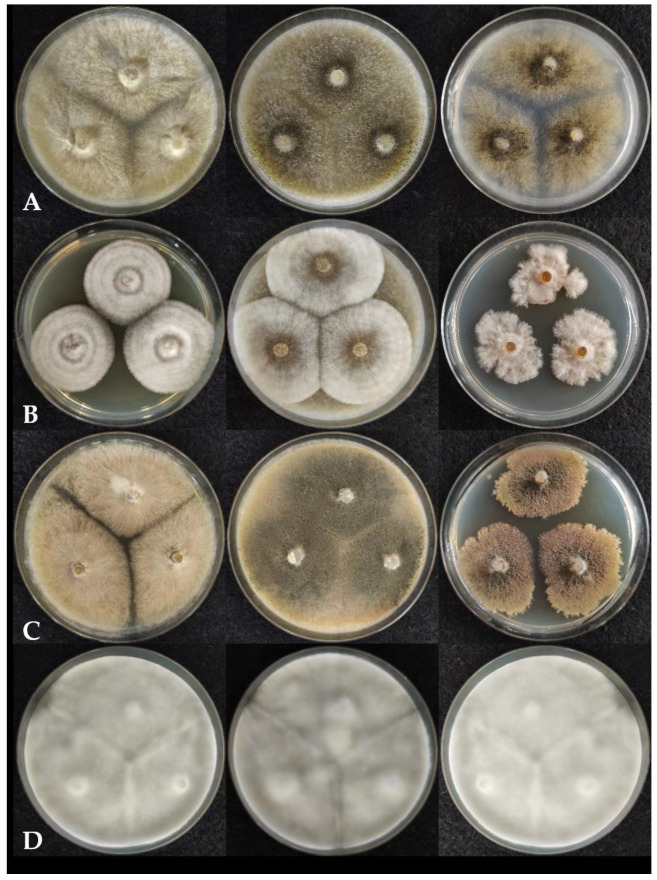
Colony morphology of *Chaetomium* strains on different agar media left to right: MEA, OA and PDA after seven days of incubation. (**A**). *Ch. cochliodes* SZMC 22473, (**B**). *Ch. interruptum* SZMC 23937, (**C**). *Ch. globosum* SZMC 23266, (**D**). *Ch. rectangulare* SZMC 26535.

Among the previously described *Chaetomium* species, *Ch. cochliodes* colonies grew rapidly on MEA, OA, and PDA (Figure 2A) reaching 65–70 mm in diameter after 7 days at 25 °C. Hyphae were light beige on MEA, while brownish on OA and PDA, with powdery surface, undulate colony edges and without colored exudates. The strains were unable to produce ascospore-containing ascomata on MEA, while strong dark green ascospore formation was observed after 7 days on OA and PDA. *Ch. interruptum* (Figure 2B) formed white mycelium on all media, brownish exudates diffusing into the media, and did not produce spores during 7 days of culturing at 25 °C. On MEA and OA, it formed regular circular colonies, while on PDA the edges of the colonies grew irregularly. Colony diameters after 7 days were 40–45, 50–60, and 30–40 mm on MEA, OA, and PDA, respectively. *Ch. globosum* (Figure 2C) colonies overgrew both MEA and OA media in 7 days at 25 °C. On PDA the strains grew slowly, with colony diameters of 30–40 mm after 7 days and a lobate edge. No ascospores were produced on MEA medium, but greenish ascospores were produced on OA and PDA. Colonies ranged from beige (MEA, OA) to brown (PDA) in color, the surface texture was floccose or velvety, and a brownish exudate was produced on all media. *Ch. rectangulare* (Figure 2D) completely overgrew all media in 7 days at 25 °C with white, cottony mycelium and without colored exudates. No ascospores were produced under any of the conditions tested.

**Figure 3:**
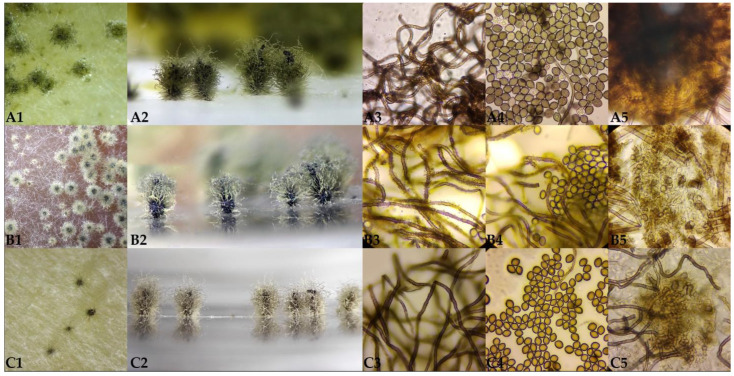
Ascomata of *Chaetomium* strains. (**A1**–**A5**): *Chaetomium cochliodes* SZMC 22473, (**B1**–**B5**): *Chaetomium globosum* SZMC 23266, (**C1**–**C5**): *Chaetomium interruptum* SZMC 23937. 1—ascomata side view; 2—ascomata top view; 3—ascomatal hairs; 4—ascospores; 5—ascomatal wall.

Ascomata of *Ch. cochliodes* (Figure 3(A1–A5)) were ostiolate, ovoid, greenish olivaceous, with brown wall, *textura intricata.* Terminal hairs were usually around the ostiolum, light brown or brown, spirally coiled, lateral hairs undulate or loosely coiled, tapering towards the tip. Mature ascospores were brown, limoniform, usually biapiculate at both ends, bilaterally flattened. Ascomata of *Ch. globosum* (Figure 3(B1–B5)) were ostiolate, greenish olivaceous, with brown wall, *textura intricata*. Terminal hairs were light brown or brown, undulate to loosely coiled, lateral hairs brown, flexuous, tapering towards the tips. Mature ascospores were greenish or brown, subglobose or limoniform, bilaterally flattened. Ascomata of *Ch. interruptum* (Figure 3(C1–C5)) were ostiolate, brown, with brown wall, *textura epidermoidea* (tissue of closely interwoven irregularly disposed hyphae without interhyphal spaces, the walls united, usually forming a membranous or epidermis-like tissue). Terminal hairs were brown undulate, lateral hairs brown, flexuous, tapering towards the tips. Mature ascospores were greenish or brown, subglobose, or limoniform, bilaterally flattened.

### 2.2. Phylogeny and Taxonomy

**Figure 4:**
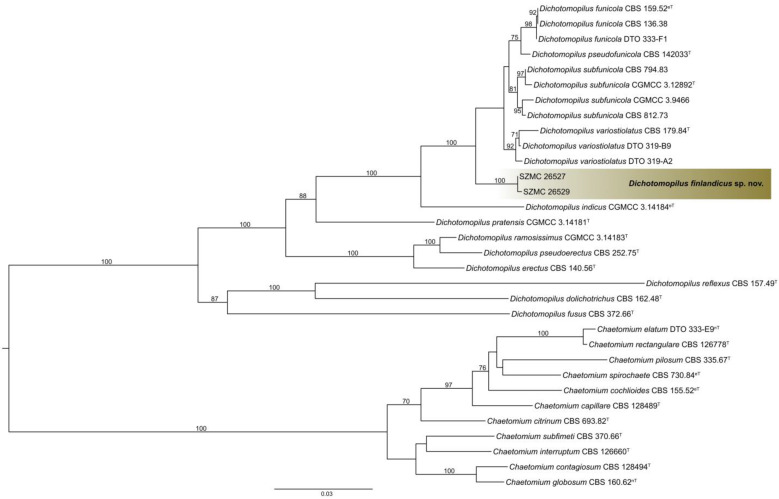
Maximum likelihood phylogeny of the genus *Dichotomopilus* inferred from the concatenated translation elongation factor 1α (*tef1α*), internal transcribed spacer (ITS), second largest subunit of RNA polymerase II (*rpb2*), and β-tubulin (*tub2*) sequences. T = ex-type, nT = ex-neotype, eT = ex-epitype. Numbers above branches are bootstrap values. Only values greater than 70% are shown.

The *tef1α, ITS, rpb2*, and *tub2* dataset consisted of 935, 639, 525, and 571 characters, respectively. The indel-based binary dataset was 100 characters long. Isolates SZMC 26527 and SZMC 26529 resolved as members of a new species with high confidence values on the phylograms obtained from both *tef1α* (Figure 1) and the other three loci (data not shown). For the final inference the four loci were concatenated and partitioned. Based on the maximum likelihood phylogenetic tree inferred from the concatenated sequences (Figure 4), isolates SZMC 26527 and SZMC 26529 formed a well-supported distinct branch inside the genus *Dichotomopilus* with the closest relatives being *D. funicola*, *D. pseudofunicola*, *D. subfunicola*, and *D. variostiolatus*. This new species is described below as *Dichotomopilus finlandicus* sp. nov.

**Figure 5:**
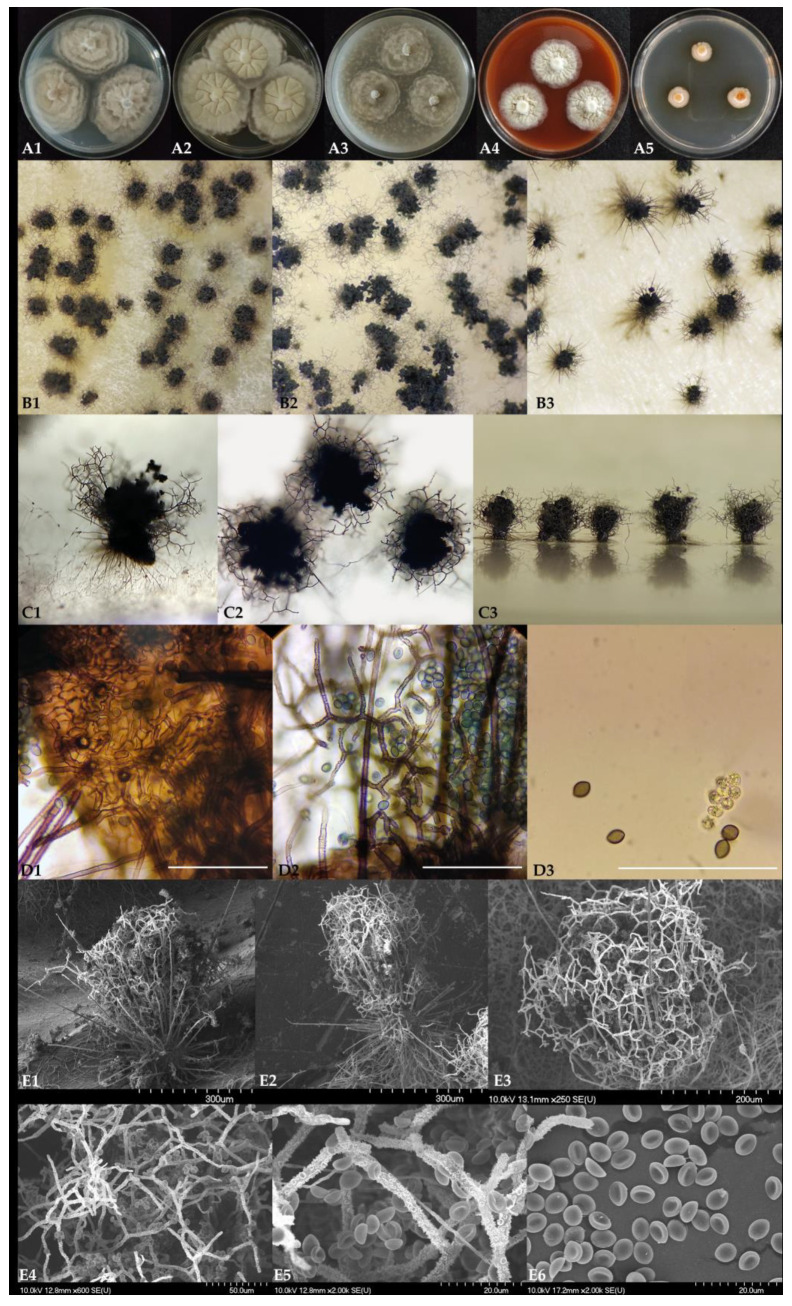
*Dichotomopilus finlandicus* SZMC 26529. (**A1**–**A5**): colony morphology of seven-day-old cultures on different agar media (A1—PDA, A2—MEA, A3—OA, A4—vegetable juice agar, A5—DG18). (**B1**–**B3**): morphology of ascomata on different agar media (left to right OA, MEA, and PDA). (**C1**–**C3**): Ascomata C1, C3—side view, C2—top view. (**D1**): ascomatal wall. (**D2**): ascomatal hairs and ascospores. (**D3**): Asci and ascospores (Bars: D1–D3 50 µm). (**E1**–**E6**): Scanning electron-microscopic images of: E1,E2: ascomata; E3: terminal ascomatal hairs; E4,E5: ascomatal hairs and ascospores; E6: ascospores.

*Dichotomopilus finlandicus* O. Kedves, S. Kocsubé, and L. Kredics sp. nov. MycoBank accession number: 840621. Etymology: Refers to the country of origin. Colonies on PDA (Figure 5(A1)) rapidly growing, about 51–54 mm in diameter after 7 days at 25 °C, with a slightly undulate edge, usually with a floccose, white to cream mycelium, irregular concentric rings, without colored exudates, and producing grey or black ascomata in 7 days. Colonies on MEA (Figure 5(A2)) rapidly growing, approximately 55–60 mm in diameter after 7 days at 25 °C with lobate edge, not forming ascospores under seven days. Colony color yellowish-white; the surface texture folded velvety to floccose. Colonies slowly growing on OA (Figure 5(A3)) at 25 °C for 7 days, about 35–38 mm in diameter with a lobate edge; with velvety surface texture. Culture color white, producing grey or black ascomata in seven days. Colonies slowly growing on vegetable juice agar media (Figure 5(A4)), about 31–33 mm in diameter over seven days at 25 °C, with a slightly undulate edge, usually with a floccose, white to cream mycelium, without colored exudates, not producing ascomata in seven days. On DG18 agar media (Figure 5(A5)) regular circular colonies showing weak growth, 12–13 mm in diameter. Colony color orange and white, producing orange exudates. *Ascomata* (Figure 5, B1–C3) superficial, ostiolate, subglobose to ovoid, dark brown, 150–180 µm high and 110–130 µm wide ascocarp. Ascomatal wall (Figure 5(D1)) comprising of brown, elongated, or irregular cells *(textura intricata).* Terminal hairs (Figure 5(C1–C3,D1)) usually around the ostiolum, light brown, or olivaceous brown, dichotomously branched 4–6 times, up to 250–320 µm long, 3–4.5 µm in diameter at the base, at wide angles and starting primarily from the upper half part, verrucose, regularly septate. Lateral hairs unbranched, seta-like, tapering towards the tip. Asci (Figure 5(D3)) fasciculate, clavate and long stipitate, stalked, 8 irregularly-arranged ascospores, spore-bearing portion 18–19 × 7–8 μm, stalks 6–11 μm long, evanescent. Ascospores: brown, broadly ellipsoid or almond-shaped 5.12–6.42 (5.88) × 4.06–4.82 (4.42) × 2.31–3.58 (2.82) µm (length × width × thickness). Growth temperature: optimum 25–30 °C, minimum 15 °C, and maximum 38 °C. Specimens examined: A piece of inlet air filter (2 × 2 cm), public building, Espoo, Finland; Holotype: freeze dried culture specimen in the Szeged Microbiological Collection (SZMC) at the Department of Microbiology, Faculty of Science and Informatics, University of Szeged, Hungary, SZMC 26529; Non-sporulating strain: SZMC 26527 from a school building, Vantaa, Finland.

### 2.3. Physiological Characterization of the Isolated Chaetomium and Chaetomium-like Strains

**Figure 6:**
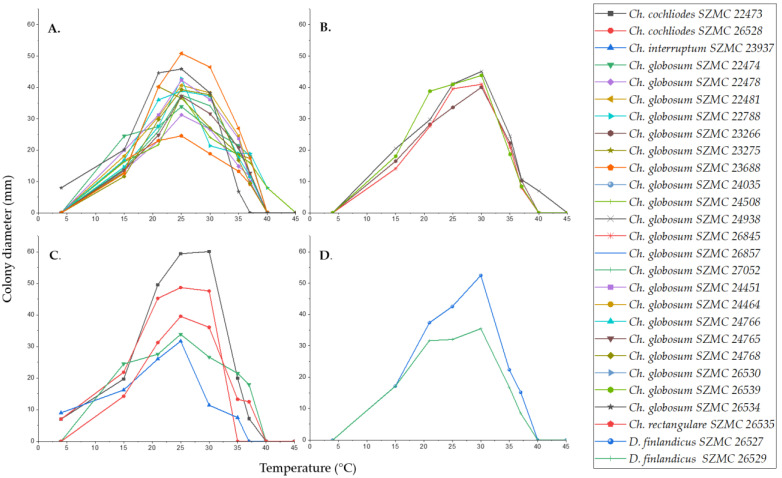
Temperature effect of growth rate: Colony diameters (cm) of the examined *Chaetomium* and *Chaetomium*-like isolates on PDA after four days measured at various temperatures ranging from 4 °C to 45 °C. (**A**): *C. globosum* strains with a temperature optimum at 25 °C. (**B**): *C. globosum* strains with a temperature optimum at 30 °C. (**C**): strains of other *Chaetomium* species. (**D**): *D. finlandicus* strains.

The optimal growth temperature of all isolates was between 25–30 °C (Figure 6). The *Ch. globosum* strains grew at temperatures between 15–40 °C, they were unable to grow at 4 °C, and only three Hungarian *Ch. globosum* isolates (SZMC 22788, SZMC 24508, and SZMC 24938) grew at 40 °C (Figure 6A,B). In addition, four Hungarian isolates of *Ch. globosum* (SZMC 23266, SZMC 24938, SZMC 26845, and SZMC 26857) showed more intensive growth at 30 °C than at 25 °C (Figure 6B). *Ch. cochliodes* SZMC 22473 and SZMC 26528, *Ch. interruptum* SZMC 23937 and *Ch. rectangulare* SZMC 26535 grew at 4 °C but among these strains *Ch. cochliodes* SZMC 26528, *Ch. interruptum* SZMC 23937 and *Ch. rectangulare* SZMC 26535 showed no growth at 37 °C, nor *Ch. cochliodes* SZMC 26528 at 35 °C (Figure 6C).

Strains of the new species *D. finlandicus* (SZMC 26527 and SZMC 26529) showed similar growth at all temperatures tested (Figure 6D). Their colony diameters were the same at 15 °C (17 mm); furthermore, at higher temperatures, strain SZMC 26527 showed a slightly higher growth. However, the optimum growth temperature for both strains was around 30 °C, and both could also grow at 37 °C.

**Figure 7:**
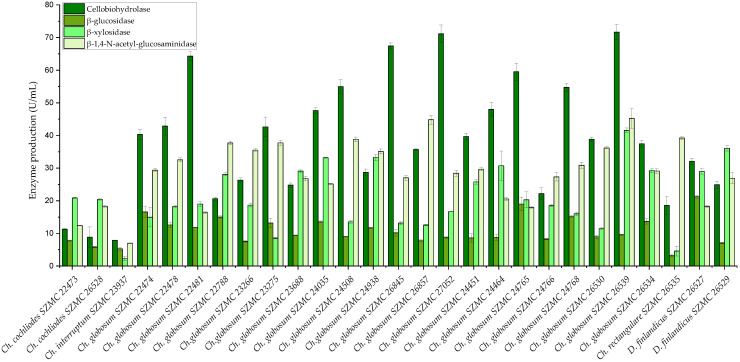
Extracellular enzyme activities of the examined *Chaetomium* and *Chaetomium*-like strains. Error bars show standard deviation of three replicates.

As *Chaetomium* and *Chaetomium*-like species are known as cellulolytic fungi, the polysaccharide (cellulose, hemicellulose, and chitin) degrading ability of the isolated strains was determined. The examined *Chaetomium* and *Dichotomopilus* strains showed various enzyme activities (Figure 7). *Ch. globosum* strains produced the highest amounts of extracellular enzymes, ranging from 20.64 to 71.67 U/mL of cellobiohydrolase, 7.57 to 18.99 U/mL of β-glucosidase, 8.54 to 41.57 U/mL of β-xylosidase, and 16.38 to 45.22 U/mL of β-1,4-N-acetyl-glucosaminidase activity. Two strains of *Ch. globosum* (SZMC 27052 and SZMC 26539) showed the highest cellobiohydrolase enzyme activities, which were almost identical in amount (71.13 ± 2.7 and 71.67 ± 2.3 U/mL, respectively). The smallest amount of extracellular enzymes was produced by *Ch. interruptum* SZMC 23937 (7.88 U/mL cellobiohydrolase, 5.29 U/mL β-glucosidase, 2.28 U/mL β-xylosidase, 6.98 U/mL β-1,4-N-acetyl-glucosaminidase) and by the two *Ch. cochliodes* strains SZMC 22473 and SZMC 26528 (11.31 and 8.83 U/mL cellobiohydrolase, 7.76 and 5.83 U/mL β-glucosidase, 20.88 and 20.43 U/mL β-xylosidase, 12.41 and 18.31 U/mL β-1,4-N-acetyl-glucosaminidase, respectively). The *Ch. rectangulare* strain SZMC 26535 also produced low amounts of polysaccharide-degrading enzymes (18.57 U/mL cellobiohydrolase, 3.24 U/mL β-glucosidase, 4.58 U/mL β-xylosidase), but the β-1,4-N-acetyl-glucosaminidase enzyme activity (39.21 U/mL) was prominent. The strains of the new species *D. finlandicus* (SZMC 26527 and SZMC 26529) had lower cellobiohydrolase enzyme production (32.14 and 24.91 U/mL) than the *Ch. globosum* strains, but in the case of the other enzymes we found similar enzyme activities (21.27 and 7.06 U/mL β-glucosidase, 29.01 and 36.08 U/mL β-xylosidase, 18.29 and 26.89 U/mL β-1,4-N-acetyl-glucosaminidase) (Figure 7).

## 3. Discussion

The dominant species in this study was *Ch. globosum* in both countries in indoor environments, as also determined in several previous studies [5,7,62]. In both countries, the species *Ch. cochliodes* was found to be also common in indoor environments. *Ch. interruptum* was isolated only from Hungary, while *Ch. rectangulare* and *D. finlandicus* only from Finland. Due to the tendency of application of cellulose-based materials (e.g., wallpapers and drywalls) in modern buildings, cellulose-degrading fungi, such as Chaetomiaceae have an increasing relevance. Most indoor strains were isolated from house dust or surface samples, while isolates from air samples were relatively rare. Similar observations were made by Fogle et al. [63] based on the analysis of samplings performed in 794 buildings in Dallas. Although several theories have emerged to explain this phenomenon, further experiments are needed to clarify the dispersal strategy of these fungi indoors.

In a previous study, Salo et al. [5] tested 42 toxin-producing *Chaetomium* isolates from Finland. In addition to the most common *Ch. globosum*, three other species, *Ch. cochliodes*, *Ch. rectangulare*, and a *Chaetomium*-like species were described for the first time from Finnish buildings. In a study by Vornanen-Winquist et al. [61], unknown indoor *Chaetomium*-like strains were designated as *Dichotomophilus* sp. The molecular results presented here revealed that the *Chaetomium*-like isolate Ch1/tu (SZMC 26529) in Salo et al. [5] and the *Dichotomopilus* sp. isolate C5/LM (SZMC 26527) from Vornanen-Winquist et al. [61] belong to the same new, previously undescribed species of the genus *Dichotomopilus*. Strain Ch1/tu was isolated from an inlet air filter and suggested to originate from the outdoor air [5], while strain C5/LM was isolated from an exhaust air filter. This may indicate that C5/LM had a possible indoor source. On the other hand, the fact that this new species was detected in both inlet and outlet air filters may also suggest that the strains were already incorporated into the filter material during production. Contamination of gypsum wall board with *Chaetomium* strains during production has been described by Andersen et al. [64].

The species *D. finlandicus* described in the recent study could be morphologically and molecularly differentiated from related species, the results of the phylogenetic analyses of the combined dataset of ITS, *tef1α*, *rpb2*, and *tub2* (Figure 4) was 100% bootstrap support. In addition, the phylogenetically closest relative species *D. funicola*, *D. pseudofunicola*, *D. subfunicola*, *D. variostiolatus*, and *D. indicus* are morphologically different from the strain we studied. Based on the morphological properties of these species studied by Wang et al. [7], ascomata, terminal hairs, and the asci were different while the shape and the size of ascospores were similar to *D. finlandicus*, which we describe here as a new species.

The enzymatic activity of the *Chaetomium* and *Chaetomium*-like strains proved to be diverse, and no correlation was found with either the isolation site or the growing substrate. These results are consistent with the findings of Abdel Azeem et al. [22], that enzyme production is isolate-dependent. The authors concluded that enzyme production has no detectable association with ecology, however, although this may be true in the case of plant host specificity, we suggest the ability to produce cellulolytic enzymes as a clear ecological advantage in the case of fungal growth on cellulose-based building materials.

In the rapid screening assays described by Salo et al. [5] and Vornanen-Winquist et al. [65], *Dichotomopilus* strains gave weaker responses than the *Ch. globosum*, *Ch. cochliodes* and *Ch. rectangulare* strains. However, strain Ch1/tu (SZMC 26529), which was designated here as the type strain of the newly described species *D. finlandicus*, inhibited boar sperm motility after 3 d of exposure, indicating that the strain produced a bioactive agent possibly affecting mitochondrial functions, or ion homeostasis [66]. Purification and identification of this substance and characterization of its biological activities will be the subject of further research.

## 4. Materials and Methods

### 4.1. Sample Collection and Isolation

*Chaetomium* and *Chaetomium*-like strains were collected and isolated from schools, flats, and offices in Finland as described previously by Salo et al. [5], as well as from houses, flats, and offices in Hungary (Table 1). To collect fungi from walls, visible colonies, or wet surfaces detected by moisture meter (Greisinger GMI 15) were sampled with sterile swabs. House dust samples were also collected with swabs. Samples were spread directly onto malt extract agar (MEA) supplemented with 2% chloramphenicol, Dichloran - Rose Bengal Agar, or Casitone Agar on site. To collect airborne fungi, air samples of 100 L were collected at 150 cm a.g.l. with 400-hole one-stage Andersen samplers [67] (MAS 100, EMD Millipore, Merck, Darmstadt, Germany; SAS IAQ, International PBI SpA, Milan, Italy; Samp l’Air MK2, AES Chemunex, Bruz, France), at a flow of 100 L/min onto MEA. Between samplings, the devices were sterilized with ethanol (abs.). Incubation of the samples was performed for 5 to 7 days at room temperature. The isolated pure cultures were deposited in the Szeged Microbiology Collection (SZMC, http://szmc.hu), Szeged, Hungary.

### 4.2. Morphological Characterization

The morphology and colony characteristics of the isolates were examined on three different media: 2% (*w*/*v*) MEA (VWR, Debrecen, Hungary), 3% (*w*/*v*) oatmeal agar (OA, Merck, Darmstadt, Germany), and potato dextrose agar (PDA, VWR, Debrecen, Hungary), and incubated for seven days in the dark at 25 °C [2,7]. Microscopic studies were performed using light—(Zeiss Primostar, Carl Zeiss, Suzhou, China), stereo—(Zeiss Stemi 305, Carl Zeiss, Suzhou, China), and scanning electron microscopes. SEM samples were prepared by stabilization in 0.1 M phosphate buffer (pH 7.3) containing 2.5% glutaraldehyde (12 h, 4 °C). The samples were then dehydrated with ethanol-water, gradually increasing the volume ratio of ethanol (50% *v*/*v*, 60% *v*/*v*, 70% *v*/*v*, 80% *v*/*v*, 90% *v*/*v*, 95% *v*/*v*, 100% *v*/*v*). Finally, the samples were dried (3 h, 30 °C) and coated with gold for microscopic examination. Electron microscopy images were taken with a 10 kV accelerating voltage Hitachi S-4700 Type II FE-SEM microscope, observing secondary electrons with magnitudes of 150×, 250×, 600×, and 2000× [68].

### 4.3. DNA Extraction, Identification, and Phylogenetic Analysis

Pure cultures of fungi were grown in 2% (*w*/*v*) MEA for 7 days at room temperature. Fungal genomic DNA was then extracted using the E.Z.N.A.®Fungal DNA Mini Kit (Omega Biotek, Norcross, GA, USA). The extracted genomic DNA was amplified by PCR with the primers listed in Table 2. The PCR mixture (20 μL) contained 2 μL 10× DreamTaq Buffer with 20 mM MgCl_2_, 2 μL of 2 mM dNTP mix, 4 μL of each primer (100 μM), 7 μL bidistilled water, 0.1 μL of 5 U/μL DreamTaq DNA Polymerase (Thermo Fischer Scientific, Vilnius, Lithuania) and 1 μL genomic DNA. Amplifications were performed in a Doppio Gradient 2 × 48-well thermal cycler (VWR International, Debrecen, Hungary) according to the amplification cycles shown in Table 2. PCR products were purified using NucleoSpin™ Gel and PCR Clean-up Kit (Macherey-Nagel, Düren, Germany). Sequencing was performed on the sequencing platform of Eurofins Genomics (http://www.eurofinsgenomics.com, accessed on 2 September 2021). The resulting sequences were submitted to the GenBank Nucleotide database (ncbi.nlm.nih.gov) under the accession numbers listed in Table 1. In addition to the sequences generated in this study, sequences of reference strains were obtained from the GenBank Nucleotide database (Table 1).

Sequences of the two *Dichotomopilus* isolates were aligned with publicly available sequences of 12 and 11 previously described *Dichotomopilus* and *Chaetomium* species, respectively. Phylogenetic analyses were conducted using four loci (ITS, *tef1α*, *rpb2*, and *tub2*). 

Sequences were aligned with Prank v170427 [69]. Alignments of the four loci were concatenated and partitioned. *Tef1α* and *rpb2* sequences were defined as two single partitions, while the *tub2* dataset was partitioned to exons and introns. The ITS dataset was divided to rDNA and ITS1-ITS2 regions. Alignments of *tub2* and ITS datasets contained relative high number of indels, therefore gaps were coded as absence/presence characters by 2matrix v1.0 [70] using the simple indel coding algorithm [71]. The two indel matrices were concatenated and added as a single partition to the dataset. Best fitting model for the phylogenetic inference was selected by using ModelTest-NG v0.1.4 [72], based on the Bayesian information criterion [73], with discrete gamma rate categories. Best fit models for each partition are shown in Table 3. Maximum likelihood analysis was performed using RAxML-NG v0.9.0 [74]. Statistical support of the best ML tree was obtained with 1000 bootstrap replicates.

### 4.4. Enzyme Production

For enzyme activity measurements, fungal strains were grown in cellulose-containing broth (20 g/L mannitol, 10 g/L KH_2_PO_4_, 5 g/L NaNO_3_, 2 g/L MgSO_4_·7H_2_O, 20 g/L cellulose powder) for seven days at 25 °C with shaking (150 rpm) on a MaxQ 8000 Incubated Stackable Shaker (Thermo Fisher Scientific, Waltham, USA). Enzyme activity measurements were performed from culture supernatants with the chromogenic substrates 4-nitrophenyl-β-D-glucopyranoside (β-glucosidase), 4-nitrophenyl-β-D-cellobiose (cellobiohydrolase), 4-nitrophenyl-β-D-xylopyranoside (β-xylosidase), 4-nitrophenyl-N-acetyl-β-D-glucosamine (β-1,4-N-acetyl-glucosaminidase) (Sigma Aldrich, St. Louis, MO, USA). 100 µL of the culture supernatants were pipetted into the wells of a microtiter plate and 100 µL of 3 mM 4-nitrophenyl substrate was added. The mixtures were incubated at 37 °C for 1 hour; thereafter the reactions were stopped by the addition of 100 μL Na_2_CO_3_ solution (0.1 M). The released 4-nitrophenol was measured on a Spectrostar Nano microtiter plate reader (BMG Labtech, Ortenberg, Germany) at 405 nm. The optical density values obtained were converted to units: 1 unit of enzyme activity was defined as the amount of enzyme required to release 1 µmol of p-nitrophenol per min under the determined reaction conditions. Calibration curve was prepared using standard solutions of 4-nitrophenol of known concentration.

### 4.5. Temperature Profiling

Optimal growth temperature ranges were determined for all *Chaetomium* and *Chaetomium*-like isolates. PDA plates were inoculated with 7 mm agar plates taken from the edge of seven-day-old colonies. The plates were incubated at 4, 15, 21, 25, 30, 35, 37, 40, and 45 °C, with six replicates each. Colony diameters were measured after four days.

## Figures and Tables

**Table 1:** *Chaetomium* and *Chaetomium*-like isolates, reference strains and their sequences involved in the study.

Species	Collection Number	Lab Code	Location of Isolation	GenBank Accession Number			
				*tef1α*	ITS	*rpb2*	*tub2*
Ac. globosum *	CBS 332.67 T		Rhizosphere, Lucknow, India	KM655479			
Ac. strumarium *	CBS 333.67 T		Soil, Lucknow, India	KC503252			
Ch. afropilosum *	CBS 145.38 T		Unknown	KT214713			
Ch. angustispirale *	CBS 137.58 T		*Fraxinus* sp., Tellerman forest, Baleshev region, Russia	KF001734			
Ch. ascotrichoides *	CBS 113.83		*Gossypium humitectum,* Argentina	KF001742			
Ch. capillare *	CBS 128489 T		Animal hair, California, USA	KT214724	NR_144860	KT214686	KT214760
Ch. cervicicola *	CBS 128492 T		Neck of *Homo sapiens* Texas, USA	KT214697			
Ch. citrinum *	CBS 693.82 T		Rice field soil, Tochigi, Japan	KT214730	NR_144863	KT214691	KT214764
Ch. coarctatum *	CBS 162.62 T		Seed of *Cappanula medium*, St. Petersburg, Russia,	KF001712			
***Ch. cochliodes***	**SZMC 22473**	**T364**	**Wall (swab), room, apartment Tatabánya, Hungary**	**MW556657**			
***Ch. cochliodes***	**SZMC 24764**	OT7 **	**Settled dust, office, Helsinki, Finland**	**MT498103**			
***Ch. cochliodes***	**SZMC 26528**	OT7b **	**Settled dust, office, Helsinki, Finland**	**MT498102**			
Ch. cochliodes *	CBS 155.52 T		Animal dung, USA	KF001721	NR_151835	KF001811	KC109772
Ch. contagiosum *	CBS 128494 T		Cornea of *Homo sapiens*, Northeast USA	KT214694	NR_144846	KT214659	KT214732
Ch. cucumericola *	CBS 378.71 T		Izmir, Turkey	KT214718			
Ch. elatum *	CBS 374.66 T		Decomposing leaf, Aptos, California, USA	KF001730			
Ch. elatum *	CBS 142034 T		Cardboard, Denmark		KX976612	KX976832	KX976954
Ch. fimeti *	CBS 139034 T		Soil, Germany	KT214698			
Ch. globosporum *	CBS 108.83 T		Green leaf of *Triticum aestivum*, Germany	KF001735			
***Ch. globosum***	**SZMC 22474**	**T365**	**Air, hotel, Budapest, Hungary**	**MW556658**			
***Ch. globosum***	**SZMC 22478**	**T369**	**Air, shop, Szentendre, Hungary**	**MW556659**	**MW541923**		
***Ch. globosum***	**SZMC 22481**	**T372A**	**House dust, basement, flat, Budapest, Hungary**	**MW556660**			
***Ch. globosum***	**SZMC 22788**	**T428B**	**Ceiling (swab), apartment, Budapest, Hungary**	**MW556661**			
***Ch. globosum***	**SZMC 23266**	**T457D**	**Under ceramic tiles in a kitchen (swab), apartment, Csepel, Hungary**	**MW556662**			
***Ch. globosum***	**SZMC 23275**	**T459A**	**Air, from gypsum board wall of a children’s room, house, Budapest, Hungary**	**MW556663**			
***Ch. globosum***	**SZMC 23688**	**T499**	**House dust (swab), living room, apartment, Budapest, Hungary**	**MW556664**			
***Ch. globosum***	**SZMC 24035**	**T536A**	**Wall, kitchen, apartment, Budapest, Hungary**	**MW556665**			
***Ch. globosum***	**SZMC 24451**	**C13/LM**	**Exhaust air filter, school, Vantaa, Finland**	**MW556666**			
***Ch. globosum***	**SZMC 24455**	C22/LM **	**Exhaust air filter, school, Vantaa, Finland**	**MT498109**			
***Ch. globosum***	**SZMC 24456**	MH5 **	**Settled dust, public building, Espoo, Finland**	**MT498108**			
***Ch. globosum***	**SZMC 24464**	MÖ9 **	**Settled dust, piggery, Orimattila, Finland**	**MT498106**	**MW541924**		
***Ch. globosum***	**SZMC 24508**	**T582D**	**Garage wall (swab), apartment, Budapest, Hungary**	**MW556667**			
***Ch. globosum***	**SZMC 24765**	**2c/26**	**Settled dust, apartment, Vantaa, Finland**	**MW310244**			
***Ch. globosum***	**SZMC 24766**	2b/26 **	**Settled dust, apartment, Vantaa, Finland**	**MT498110**			
***Ch. globosum***	**SZMC 24768**	**C22**	**Settled dust, apartment Vantaa, Finland**	**MW556668**			
***Ch. globosum***	**SZMC 24769**	MH52 **	**Settled dust, public building, Espoo, Finland**	**MT498107**			
***Ch. globosum***	**SZMC 24938**	**626C**	**Wall (swab), living room, house, Kazincbarcika, Hungary**	**MW556670**			
***Ch. globosum***	**SZMC 26530**	Ruk10 **	**Settled dust, apartment, Vantaa, Finland**	**MT498101**	**MW541927**		
***Ch. globosum***	**SZMC 26534**	MTAV35 **	**Settled dust, University of Oulu, Finland**				
***Ch. globosum***	**SZMC 26539**	**3b/APP**	**Exhaust air filter, public building** **, Espoo, Finland**	**MW588207**			
***Ch. globosum***	**SZMC 26845**	**T706**	**Wall (swab), kitchen, apartment, Budapest, Hungary**	**MW556672**			
***Ch. globosum***	**SZMC 26857**	**T711**	**Wallpaper (swab), living room, apartment, Budapest, Hungary**	**MW556673**			
***Ch. globosum***	**SZMC 27052**	**T730A**	**On paper packaging, imported from Sri Lanka, swab factory, Pécs, Hungary**	**MW556674**			
Ch. globosum *	CBS 160.62 T		Compost, Germany	KT214704	NR_144851	KT214666	KT214742
Ch. globosum *	MUCL 39526 T		Dead stem of *Juncus* sp., Hungary	KF001710			
Ch. globosum *	CBS 666.82		Unknown		KX976617	KX976833	KX976959
Ch. graminiforme *	CBS 506.84 T		*Acer* sp., Muskoka District, Ontario, Canada	KT214725			
Ch. grande *	CBS 126780 T		Leaf of *Triticum aestivum*, Naghadeh, Iran	KT214692			
***Ch. interruptum***	**SZMC 23937**	**T531B**	**Under wallpaper (swab), office, Budapest, Hungary**	**MW588206**	**MW301425**		
Ch. interruptum *	CBS 126660 T		Seed of *Triticum aestivum*, Hadishahr East Azerbaijan Province, Iran	KT214703	KT214564	KT214665	
Ch. madrasense *	CBS 315.74 T		Rhizosphere of *Pennisetum typhoides*, Chennai, Tamil Nadu, India	KF001741			
Ch. novozelandicum *	CBS 124555 T		Dead decaying twig, Otaki, New Zealand	KT214715			
Ch. pilosum *	CBS 335.67 T		Grain of *Triticum aestivum*, Perth, Western Australia	KT214729	NR_144862	FJ666387	KT214763
Ch. pseudocochliodes *	CGMCC 3.9441 T		Roots of *Panax notoginseng*, Wenshan, Yunnan Province, China	KF001726			
Ch. pseudoglobosum *	CBS 574.71 T		Unknown	KT214712			
***Ch. rectangulare***	**SZMC 26531**	MO13 **	**Settled dust, piggery, Orimattila, Finland**	**MT498104**	**MW541928**		
***Ch. rectangulare***	**SZMC 26535**	MO15 **	**Settled dust, piggery, Orimattila, Finland**	**MT498105**	**MW541929**		
Ch. rectangulare *	CBS 126778 T		Leaf of *Hordeum vulgare*, Salmas, West Azerbaijan province, Iran	KT214726	NR_144817	HM365285	KT214688
Ch. spiculipilium *	CBS 373.66 T		Decaying vegetable debris, California, USA	KF001719			
Ch. spirochaete *	CBS 730.84 T		Animal dung, Great Smokey Mountains, Tennessee, USA	KF001729	NR_144823	KF001819	JN256191
Ch. subaffine *	CBS 637.91 T		Cereal, USSR	KF001727			
Ch. subfimeti *	CBS 370.66 T		Paper and vegetable material, Cardiff, Wales	KT214701	NR_144850	FJ666385	KT214739
Ch. subglobosum *	CBS 149.60 T		Dead herbaceous stem, St. Petersburg, Russia	KF001718			
Ch. telluricola *	CBS 151.59 T		Soil, Suffolk, Lakenheath Warren, United Kingdom	KT214723			
Ch. tenue *	CBS 139.38 T		Unknown	KT214707			
Ch. umbonatum *	CBS 293.83 T		Soil, Nova Scotia, Canada	KT214714			
Ch. undulatulum *	CBS 126775 T		Leaf of *Hordeum vulgare*, Bonab, East Azerbaijan province, Iran	KT214720			
Ch. unguicola *	CBS 128446 T		Nail of *Homo sapiens*, Los Angeles, USA	KT214706			
Ch. megalocarpum *	CBS 149.59 T		Leaf of *Ficus carica*, Greece	KF001738			
D. dolichotrichus *	CBS 162.48 T		USA	KC485023	HM449049	KX976852	JF772462
D. erectus *	CBS 140.56 T		*Petroselinum sativum*, USA	KC485018	HM449044	KX976854	JF772458
D. funicola *	CBS 159.52 T		Germany	KC485013	GU563369	KX976856	JF772461
D. funicola *	CBS 136.38		Unknown			KX976857	
D. funicola *	DTO 333-F1		Dust, outdoors, Denmark		KX976658	KX976858	KX977000
D. fusus *	CBS 372.66 T		Unknown	KM655463	KM655333	KX976859	KX977002
D. indicus *	CGMCC 3.14184 T		Rhizosphere of *Panax Notoginseng*, Wenshan county, Yunnan Province	KC485005	GU563367	KX976861	JF772453
D. pratensis *	CGMCC 3.14181 T		Soil, Huangnan, Qinghai Province, China	KC485017	GU563372	KX976866	JF772450
D. pseudoerectus *	CBS 252.75 T		Air, Uttar Pradesh, India		NR_147674	KX976869	KX977009
D. pseudofunicola *	CBS 142033 T		Dust, USA		KX976668	KX976870	KX977010
D. ramosissimus *	CGMCC 3.14183 T		Rhizosphere of *Panax notoginseng*, Wenshan county, Yunnan Province, China	KC485021	GU563371	KX976871	JF772452
D. reflexus *	CBS 157.49 T		Germinating seed, USA	KC485027	HM449051	KX976873	JF772460
D. subfunicola *	CGMCC 3.12892 T		Soil, Shihezi, Xinjiang Autonomous Region, China	KC485014	JX867125	KX976875	JX867122
D. subfunicola *	CGMCC 3.9466		Rhizosphere of *Panax notoginseng*, Yunnan, China	KC485016	GU563368	KX976876	JF772446
D. subfunicola *	CBS 794.83		Paper, Switzerland		GU563368	KX976876	KX977013
D. subfunicola *	CBS 812.73		Pistol belt, New Guinea		KX976670	KX976877	KX977012
D. variostiolatus *	CBS 179.84		Tarpaulin, New Guinea		NR_147676	KX976879	KX977014
D. variostiolatus *	DTO 319-B9		Dust, Thailand		KX976674	KX976881	KX977016
D. variostiolatus *	DTO 319-A2		Dust, USA		KX976673	KX976880	KX977015
***Dichotomopilus* sp.**	**SZMC 26527**	**C5/LM**	**Exhaust air filter, school, Vantaa, Finland**	**MW556671**	**MW541925**	**MZ665530**	**MZ665528**
***Dichotomopilus* sp.**	**SZMC 26529**	Ch1/tu **	**Inlet air filter, public building, Espoo, Finland**	**MT644127**	**MW541926**	**MZ665531**	**MZ665529**

Strains isolated during this study are set in bold. * reference strain [2,7], ** described in [5].

**Table 2:** List of the amplified genes, used primers, and PCR conditions.

Gene	Primer	PCR Condition	
*tef1α*	EF1-728F: CATCGAGAAGTTCGAGAAGG TEF1-LLErev: AACTTGCAGGCAATGTGG	94 °C 5 min 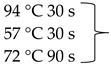 72 °C 7 min	40 cycles
ITS	ITS1: TCCGTAGGTGAACCTGCGG ITS4: TCCTCCGCTTATTGATATGC	94 °C 2 min 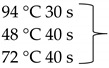 72 °C 2 min	35 cycles
*tub2*	BT2a: GGTAACCAAATCGGTGCTGCTTTC BT2b: ACCCTCAGTGTAGTGACCCTTGGC	94 °C 2 min 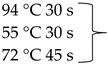 72 °C 7 min	35 cycles
*rpb2*	RPB2 5F_Eur: GAYGAYCGKGAYCAYTTCGG RPB2 7CR_Eur: CCCATRGCYTGYTTRCCCAT	94 °C 5 min	
		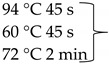	5 cycles
		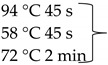	5 cycles
		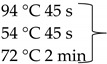	30 cycles
		72 °C 7 min	

**Table 3:** Best-fit models for each partition proposed by ModelTest-NG based on Bayesian information criterion.

Partition	Best-Fit Model
*rpb2*	TrN + G4
*tef1α*	TIM2 + G4
*tub2* intron	HKY + G4
*tub2* exon	TrN + G4
rDNA	F81 + G4
ITS	TIM2 + G4
Indel	BIN + ASC_LEWIS

## Data Availability

Nucleotide sequences were deposited in the GenBank Nucleotide Database (https://www.ncbi.nlm.nih.gov), accession numbers are provided in Table 1.
